# A Novel Algorithm for Detecting Pedestrians on Rainy Image

**DOI:** 10.3390/s21010112

**Published:** 2020-12-27

**Authors:** Yuhang Liu, Jianxiao Ma, Yuchen Wang, Chenhong Zong

**Affiliations:** College of Automobile and Traffic Engineering, Nanjing Forestry University, Nanjing 210037, China; liuyh13376098819@163.com (Y.L.); 15250989036@163.com (Y.W.); 15295516908@163.com (C.Z.)

**Keywords:** intelligent traffic system, deep learning, pedestrian detection, de-raining processing

## Abstract

Pedestrian detection is widely used in cooperative vehicle infrastructure systems. Traditional pedestrian detection methods perform sufficiently well under sunny scenarios and obtain trustworthy traffic data. However, the detection drastically decreases under rainy scenarios. This study proposes a pedestrian detection algorithm with a de-raining module that improves detection accuracy under various rainy scenarios. Specifically, this algorithm determines the density information of rain and effectively removes rain streaks through the de-raining module. Then the algorithm detects pedestrians as a pair of keypoints through the pedestrian detection module to solve the problem of occlusion. Furthermore, a new pedestrian dataset containing rain density labels is established and used to train the algorithm. For the scenarios of light, medium, and heavy rain, extensive experiments on synthetic datasets demonstrate that the proposed algorithm increases AP (average precision) of pedestrian detection by 21.1%, 48.1%, and 60.9%. Moreover, the proposed algorithm performs well on real datasets and achieves improvements over the state-of-the-art methods, which reveals that the proposed algorithm can significantly improve the accuracy of pedestrian detection in rainy scenarios.

## 1. Introduction

Rain is a very common kind of weather in real life, it can badly affect the visibility of a camera. Especially in heavy rain, the rain streaks accumulate seriously, which result in blur and distortion in acquired images. This type of interference considerably degrades the accuracy of pedestrian detection [1,2,3]. Smart traffic has become a modern trend, the development of which countries pay a great deal of attention to. A pedestrian detection algorithm which has high accuracy is needed worldwide. For this reason, it is important to realize pedestrian detection on rainy days.

Although lots of attention is paid to the study of image precipitation and pedestrian detection [4,5], there are few systematic studies of pedestrian detection on rainy days. In the image de-raining section, Fu et al. [6] proposed a method that can de-rain some important parts in the rainy image. Yang et al. [7] designed a deep cycle expansion network to detect and remove rain streaks. However, they did not consider the scale and density of rain streaks and mainly concentrated on a specific type while there are still rain streaks left in the processed images [8]. Therefore, more efficient and robust methods are needed to process the images captured in different rain conditions. At the same time, the existing synthetic datasets lack the scale and density of rain streaks corresponding to each synthetic rainy image, and there are few pedestrian targets in the image. These datasets cannot meet the requirement of training pedestrian detection algorithm on rainy days. For example, Fu et al. [6] and Yang et al. [7] synthesized a novel large-scale dataset consisting of rainy images and they trained a single network based on this dataset for image de-raining. However, one drawback of this approach is that a single network may not be qualified to achieve a good de-raining effect without considering multiple rainfall scenarios.

In the pedestrian detection section, existing advanced pedestrian detection algorithms—such as faster regions with convolutional neural network (Faster-RCNN), you only look once v3(YOLOv3), deconvolutional single shot detector (DSSD), etc.—are mostly based on anchor boxes. These models are too complex to improve the detection speed. Moreover, on rainy days pedestrians are often different because most pedestrians wear raincoats or carry umbrellas. Thus there are a lot of occlusions among pedestrians.

To solve these problems, we propose a pedestrian detection algorithm for the scenario of rainy days. The proposed algorithm includes a de-raining module and pedestrian detection module. Specifically, the de-raining module injects the Recurrent Neural Network into Generative and Discriminative Network. It can determine the density information of rain, find the region where the rain streaks and its surrounding structures, and effectively removes rain streaks. Then the pedestrian detection module introduces a one-stage detector CornerNet-Lite without anchor boxes and detects pedestrians as a pair of keypoints to solve the problems of occlusions. Finally, we realize pedestrian detection on rainy days.

Furthermore, we synthesize a rainy dataset for pedestrian detection, consisting of light, medium, and heavy rain. Then we employ the proposed algorithm to test the synthetic dataset and the real dataset, both of which achieve excellent results.

Overall, one of our contributions is the injection of the recurrent neural network into the generative and discriminative network, which is novel and works effectively in removing rain streaks. Our other main contribution is to introduce CornerNet-Lite to solve the problems of occlusions among pedestrians, and modified the loss function of the algorithm to improve the detection accuracy, as shown in our experiments in Section 4. Our final contribution is to establish a new pedestrian dataset containing various rain density labels and used it to train and test our algorithm.

This paper is organized as follows: Section 2 introduces some related works; Section 3 explains the proposed algorithm; The results of the proposed algorithm on the synthetic dataset and the real dataset are analyzed on Section 4; and finally, conclusions and some future works are discussed in Section 5.

## 2. Related work

### 2.1. Single Image De-Raining Based Methods

Because there is no time information in a single image, a single image de-raining is more challenging. For this task, the widely used traditional methods include dictionary learning [9], Gauss hybrid model (GMMs) [10], and low rank representing [11]. Kang et al. [12] used a bilateral filter to decompose an image into the low-frequency and high-frequency parts. Built upon a non-linear generative model of the rainy image, Luo et al. [13] proposed a dictionary learning based algorithm for single image de-raining. Gu et al. [14] proposed a joint convolutional analysis and synthesis (JCAS) sparse representation model and used the global information to de-rain. Chang et al. [15] analyzed the rainy and clean image in both local gradient and nonlocal domain, and proposed a compositional directional total variational and low-rank prior.

In recent years, deep learning is gradually applied to image de-raining. Fu et al. [6] used a priori image domain knowledge by focusing on high frequency detail and de-rained, which removes background interference and focuses the model on the structure of rain in images. Yang et al. [7] proposed a recurrent rain detection and removal network that removes rain streaks progressively. Zhang et al. [8] presented a multi-stream densely connected de-raining network that efficiently leverages features from different scales. It consists of a new residual-aware classifier and a multi-stream densely connected network that does not over-process or under-process the rainy image. Fu et al. [16] first introduced deep learning methods into the problem of de-raining, breaking down rainy images into low-frequency and high-frequency sections, and then mapping high-frequency portions to the rain streak layer. Yang et al. [17] designed a deep cycle expansion network to detect and remove rain streaks. Li et al. [4] used a dilated convolutions network to acquire a large receptive field, and then broke down the rain removal into multiple stages. Zhang et al. [18] proposed a conditional GAN-based framework to de-rain and introduced an improved loss function. The algorithm used local and global information to determine whether the de-raining image is true or false. Ren et al. [19] unfolded a shallow residual network (ResNet) repeatedly and proposed progressive ResNet (PRN). A recurrent layer is further introduced to exploit the dependencies of deep features across stages, forming the progressive recurrent network (PReNet). Cai et al. [20] used a residual network with only two residual blocks, which is recursively unfolded to remove rain streaks in multiple stages. Meanwhile, the two residual blocks can be recursively computed in one stage, forming the dual recursive network. However, only few studies involved the scale and density of rain streaks.

Therefore, we plan to study the de-raining of a single image from the aspects of light, medium, and heavy rain. In our method, we utilize a recurrent neural network and a generative and discriminative network as the de-raining module. Specifically, we use a recurrent neural network to find the areas in the input image that need to be focused. Then, we use a generative network to produce the most realistic image with the rain removal and use the discriminative network to evaluate the quality of the de-raining image.

### 2.2. Pedestrian Detection Methods

In terms of pedestrian detection, Girshick et al. [21] proposed R-CNN and introduced the two-stage method for the first time. The algorithm uses selective search to generate region of interest (ROI), then extracts each area from the image and processes it by the convolutional neural network. Later, Girshick et al. [22] designed ROI pooling and proposed Fast-RCNN. By introducing the regional proposal network (RPN), Ren et al. [23] proposed Faster-RCNN by employing a set of pre-set anchor boxes for detection to improve the efficiency of detection.

On the other hand, Redmon et al. [24] eliminated ROI pooling and proposed the YOLO algorithm. The algorithm introduces one kind of one-stage method and predicts bounding box coordinates directly from the image. After then, Redmon et al. [5,25] then adjust their network structure and propose YOLO9000 and YOLOv3. Fu et al. [26] used hourglass network to extract feature information and predicted bounding boxes more accurately. Lin et al. [27] developed RetinaNet to solve the problem of detection accuracy caused by the large imbalance of positive and negative anchor boxes in the one-stage method. Bochkovskiy et al. [28] combined universal features—including weighted-residual-connections, cross-stage partial connection, and cross mini-batch normalization, etc.—and new features including dropblock regularization, mosaic data augmentation, and CIoU loss, etc. and proposed yolov4. In recent years, anchor-free detectors are developed. Law et al. [29,30] proposed a new approach, called CornerNet, which introduced a new compact backbone architecture and detected pedestrians as a pair of keypoints, and finally improved accuracy at real-time efficiency. Tian et al. [31] took the advantage of all points in a ground truth bounding box to predict the boxes and use the center-ness branch to detect the low-qualities bounding boxes. All the above mentioned anchor-free methods avoid all hyper-parameters related to anchor boxes and the detection speed is improved.

However, academics few systematically study the pedestrian detection task on rainy days. Pedestrian detection on rainy days is necessary and special because most pedestrians wear raincoats or carry umbrellas. There are a lot of occlusions among pedestrians. To avoid too many algorithm parameters and realize pedestrian detection among occlusions, we detect a pedestrian as a pair of keypoints—the top-left corner and bottom-right corner of the bounding box. Then we modified the loss function in the pedestrian detection module to improve the accuracy of pedestrian detection. In Section 4, we will show some evaluations between our method and the state-of-the-art methods.

## 3. The Proposed Algorithm

**Figure 1:**
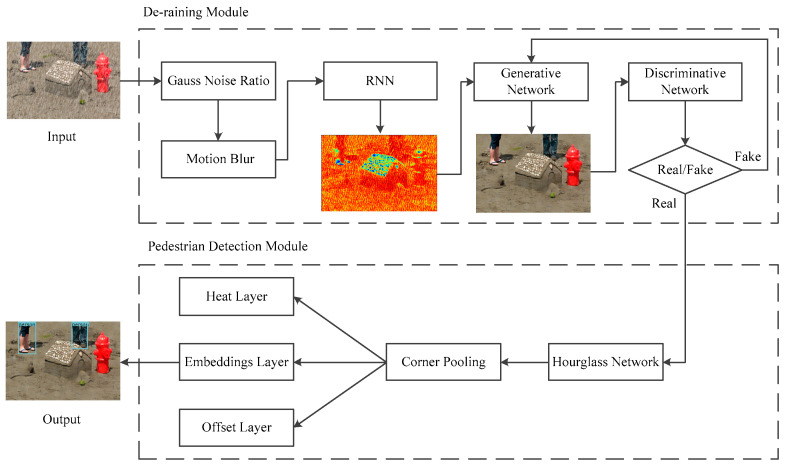
Architecture of the proposed algorithm.

The proposed algorithm includes a de-raining module and pedestrian detection module, as shown in the flow chart in Figure 1. The de-raining module is used to evaluate the level of the rain condition. Then the module uses a Recurrent Neural Network (RNN) and a Generative and Discriminative Network (GAN) to de-rain depending on the assessment results [32,33], The pedestrian detection module introduces the detector CornerNet-Lite without anchor boxes. It detects pedestrians as a pair of keypoints to solve the problems of occlusions. The details of the two modules are described below.

### 3.1. De-Raining Module

Generally, the rain streaks-removal problem is difficult, since there are two challenges involved in this task. First, the regions occluded by rain streaks are not given. Second, for most of the time, the information about the background of the occluded regions is completely lost. The problem gets worse when the rain streaks are distributed densely across the input image. To resolve the problem, this paper employed a RNN and a GAN. The module first evaluates the level of the rain condition. Then the RNN is used to find the region where the rain streaks and its surrounding structures. These regions are necessary for the generative network to focus on, so that it can generate better local image restoration, and for the discriminative network to focus the assessment on. The GAN is used to produce the de-rained image and ensure that the outputs look like the real images. The loss of the module can be expressed as

(1) $$\underset{Gen}{\min}\;\underset{Dis}{\max}E_{O\sim P_{clean}}\left[\log\left(Dis\left(O\right)\right)\right]+E_{R\sim P_{rainstreak}}\left[\log\left(1-Dis\left(Gen\left(R\right)\right)\right)\right]$$

where *Gen* is the generative network, *Dis* is the discriminative network, *R* is the image obscured by rain streaks, *O* is the original image.

#### 3.1.1. Recurrent Neural Network

**Figure 2:**
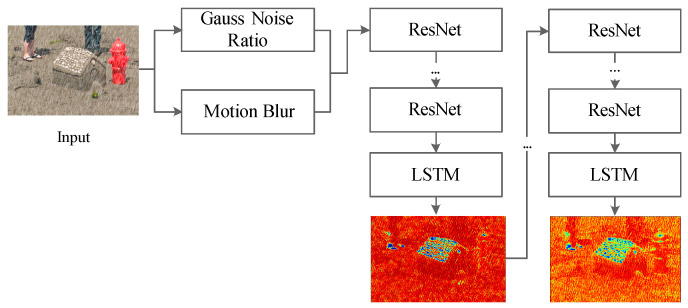
Architecture of the RNN.

For the input image, gaussian noise ratio and motion blur are used to evaluate the level of the rain condition. Then we use a recurrent neural network to find the areas in the input image that need to be focused and generate the attention map. These areas are mainly the region where the rain streaks and its surrounding structures. This method enables the generated network to better deal with the areas affected by rain streaks. In a recurrent neural network, each module consists of a five-layer residual network (ResNet) and an LSTM unit [34], as shown in Figure 2.

**Figure 3:**
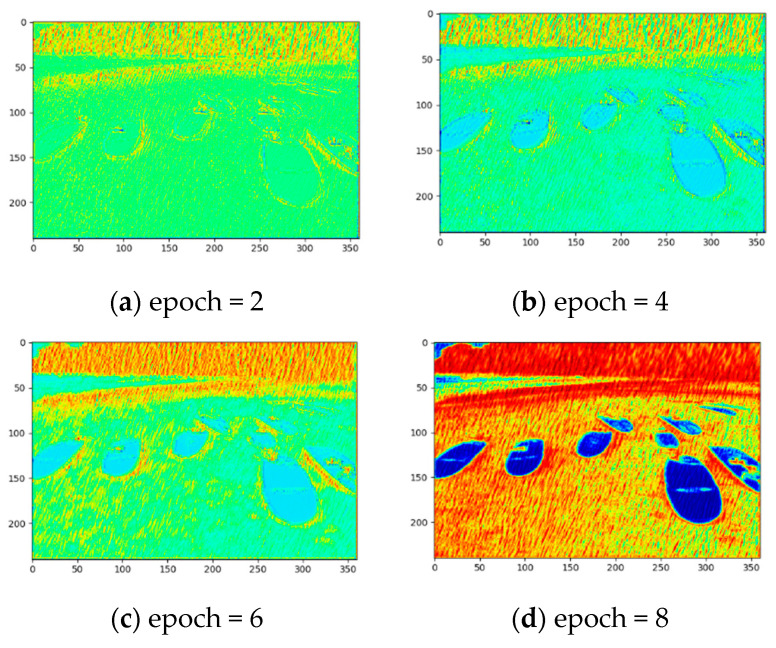
Visualization of the recurrent neural network learning process.

After several iterations, the recurrent neural network finally finds the areas of attention in the image, as shown in Figure 3. The attention map is a matrix whose elements range from zero to one, and the greater the matrix element, the more important it suggests.

Figure 3 shows the learning process of the attention map generated by our network during the training. The red streaks are the areas where need to focus on. The blue areas indicate the region where need retain background information. As can be seen, the recurrent neural network not only can find the areas covered by the rain streaks, but also tries to find the surrounding areas affected by the streaks.

#### 3.1.2. Generative and Discriminative Network

**Figure 4:**
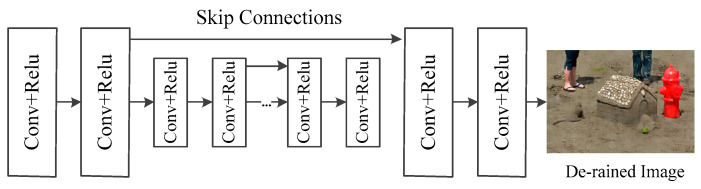
Architecture of the generative network.

The generative and discriminative network includes a generative network and a discriminative network. The generative network is used to produce the most realistic image with the rain removal. To get more contextual information, the network has 14 Conv-Relu blocks, and we add skip connections to solve the gradient disappearance problem. The specific structure is shown in Figure 4.

**Figure 5:**
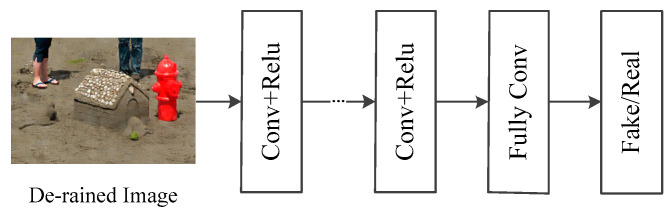
Architecture of discriminative network.

The discriminative network is used to differentiate fake images from real ones [32]. A few GAN-based methods adopt global or local image-content consistency in the discriminative part. The global discriminator looks at the whole image to check, while the local discriminator looks at small specific regions. This paper uses a local discriminator to make decisions, but the network cannot find which areas remove rain streaks. To solve this problem, we mainly focus on the attention map generated by a recurrent neural network to discover these areas. Our goal is to guide the discriminant network to focus on the areas indicated by the attention map. Specifically, the discriminant network in this paper consists of seven layers of Conv-Relu and a full connection layer, as shown in Figure 5.

### 3.2. Pedestrian Detection Module

**Figure 6:**
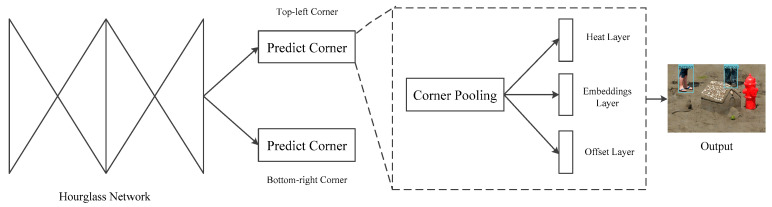
Architecture of pedestrian detection module.

Pedestrians on rainy days are special because most pedestrians wear raincoats or carry umbrellas, so there is a lot of occlusions among pedestrians. Aim at the problem of occlusion, the pedestrian detection module detects a pedestrian as a pair of keypoints—the top-left corner and bottom-right corner of the bounding box. For the images after removing rain streaks, this module first uses the hourglass networks as the backbone network to extract image features [35]. The output feature map is processed by corner pooling to obtain the position information of corners. We detect the corner position of pedestrians through two sets of heat layers and then optimize the loss of the embedded layer to make the distance between the two corners embedded into the same object smaller and smaller. At the same time, we calculate the loss of the offset layer, and adjust the position of corners to generate a more compact prediction box, as shown in Figure 6.

#### 3.2.1. Hourglass Network

**Figure 7:**
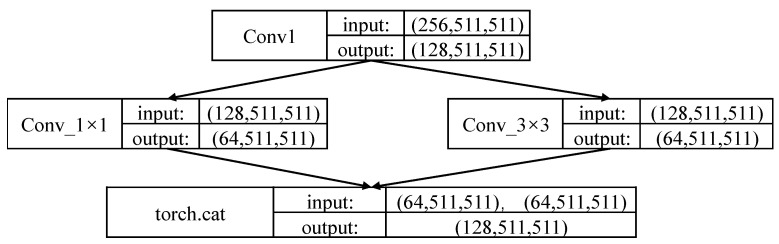
Architecture of the fire module.

This paper utilizes two stacked hourglass networks for feature extraction, and the resolution of the output feature map is 64 × 64. The hourglass network is composed of two 3 × 3 convolutional layers and a skip connected residual block. However, the traditional hourglass network has too many parameters and costs lots of computation resources. Combining with the network of SqueezeNet [36], a Fire Module is used in the hourglass network to reduce the processing amount of pixels, as shown in Figure 7. There are two main ways to reduce network complexity: (1) 1 × 1 convolution kernel is used to process the image and change the dimension of the image to reduce the network parameters. (2) An extended layer consists of a 1 × 1 and 3 × 3 convolution kernel is employed to provide the results.

#### 3.2.2. Corner Pooling

Aim at the problem of occlusion, pedestrian positions can be simplified to top-left and bottom-right points in the image. There is no rule to follow for different pedestrians’ corner position. If we use the common pooling operation, it is difficult to predict the corner position. However, the right side of the top-left corner point has the feature information of the target’s top, and the bottom side of the corner point has the feature information of the target’s left side. In this paper, we use the corner pooling network to extract the feature information of pictures and predict the location of pedestrians.

**Figure 8:**
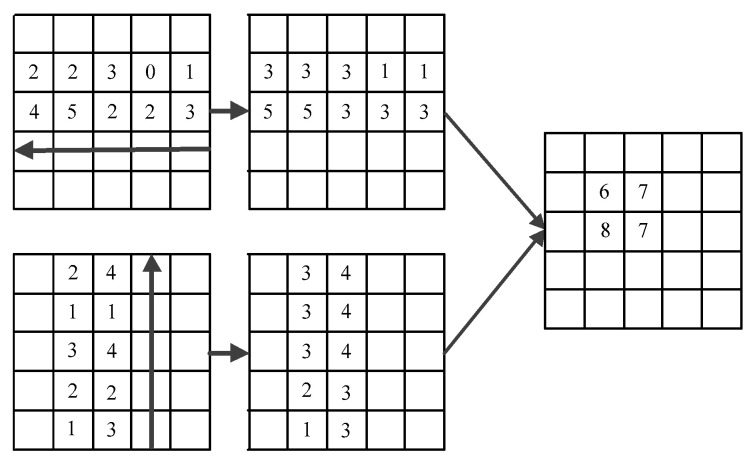
Process of corner pooling.

The corner pooling layer is used to process the feature map of hourglass networks output. Then the maximum pooling is done horizontally from right to left to obtain the feature map. At the same time, another feature map is obtained by maximum pooling from bottom to top in the vertical direction. As shown in Figure 8.

#### 3.2.3. Loss Function

The loss function of the whole algorithm can be divided into three parts, which are the loss of heat, embedded and offset layer. In order to improve the convergence speed of the overall model, we introduce Adam [37] algorithm to optimize the whole loss. Both α and β are set to 0.5

(2) $$L=L_{heat}+\alpha L_{pull}+\beta L_{push}+L_{off}$$

where *L* is the total loss of the algorithm, $L_{heat}$ is the loss of the predicted corner positions, $L_{pull}$ is the loss generated by predicting two corners of the same target, $L_{push}$ is the loss generated by predicting the two angles of different targets, $L_{off}$ is the precision information that is lost during rounding calculations.

Heat Layer—Based on focal loss [27], the loss of the heat layer is modified to predict the position of the corner:

(3) $$L_{heat}=\frac{-1}{N}\sum_{c=1}^{C}\sum_{i=1}^{H}\sum_{j=1}^{W}\left\{ \begin{array}{l} \log\left(p_{cij}\right)\begin{array}{cc} & \begin{array}{cc} if & y_{cij}=1 \end{array} \end{array}\\ {\left(1-y_{cij}\right)}^{\beta }{\left(1-p_{cij}\right)}^{a}\log\left(p_{cij}\right)\begin{array}{cc} & otherwise \end{array} \end{array}\right.$$

where $L_{heat}$ is the loss of the predicted corner positions, $p_{cij}$ is the predicted probability of channel *C* at coordinates (*i*,*j*), $y_{cij}$ is the true mark of channel *C* at coordinates (*i*,*j*), *C* is the number of channels, *H* is the height, *W* is the width, *N* is the number of objects, β = 4, a = 2.

Embedding Layer—To match the upper-left and lower-right key points of the same target, we make the loss of predicting the two corners of the same target as small as possible and the two corners of the different target as large as possible [38,39]. Then we modify the loss $L_{pull}$ and the $L_{push}$ as

(4) $$L_{pull}=\frac{1}{N}\sum_{k=1}^{N}\left[\left|e_{tk}-e_{k}\right|+\left|e_{bk}-e_{k}\right|\right]$$

(5) $$L_{push}=\frac{1}{N\left(N-1\right)}\sum_{k=1}^{N}\sum_{j=1}^{N}\max\left(0,1-\left|e_{k}-e_{j}\right|\right)$$

where $L_{pull}$ is the loss generated by predicting two corners of the same target, $L_{push}$ is the loss generated by predicting the two angles of different targets, $e_{tk}$ is the embedded vector at the top-left corner, $e_{bk}$ is the embedded vector at the bottom-right corner, $e_{k}$ is the average of $e_{tk}$ and $e_{bk}$, *N* is the number of objects, $e_{j}$ is $e_{k}$ interchange two or three columns.

Offset Layer—The hourglass networks have the processes of down-sampling and re-sampling. The network maps to the coordinates in the original image based on the rounded down-sampling coordinates. Therefore, the whole process resulting in a loss of precision [40] is

(6) $$L_{off}=\frac{1}{N}\sum_{k=1}^{N}Smooth_{L_{1}}Loss\left({ο}_{k},{\overset{⌢}{ο}}_{k}\right)$$

(7) $${\mathrm{Smooth}}_{L_{1}}\left(x\right)=\left\{ \begin{array}{ll} 0.5x^{2} & if\;\;\left|x\right|<1\\ \left|x\right|-0.5 & otherwise \end{array}\right.$$

(8) $${ο}_{k}=\left(\begin{array}{cc} \frac{x_{k}}{n}-\left\lfloor \frac{x_{k}}{n}\right\rfloor & \begin{array}{cc} , & \frac{y_{k}}{n} \end{array} \end{array}-\left\lfloor \frac{y_{k}}{n}\right\rfloor \right)$$

where $L_{off}$ is the precision information lost during rounding calculations, ${ο}_{k}$ is the offset, $x_{k}$ is the x-axis coordinate of a corner k, $y_{k}$ is the y-axis coordinate of corner k, *n* is a multiple of the down-sampling, ${\mathrm{Smooth}}_{L_{1}}$ is a loss function [23], ⌊ *x* ⌋ is the maximum integer that is not greater than *x*.

## 4. Experimental Result

### 4.1. Synthetic Dataset

**Figure 9:**
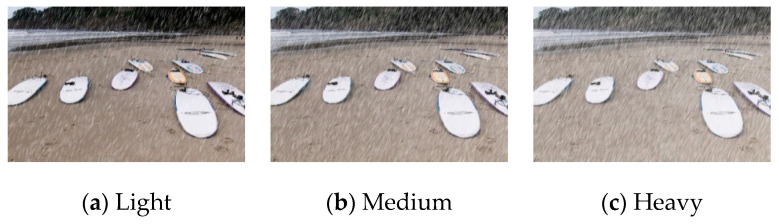
Synthetic samples in three different conditions.

Although there are many large-scale synthetic datasets [6,7,18], they lack the labels which contain information for various rain density levels, and there are few pedestrian targets in the image. Thus, this paper builds a new dataset containing pedestrian targets and rainy labels. This paper selected the COCO2014 [41] as raw dataset and extracted the pedestrian targets from it, finally build the pedestrian dataset in rainy scenario. The obtained dataset with a total of 66,808 images, in which 45,174 are regarded as training images, and the remaining 21,634 images are test set. The number of pedestrians in each image varies and the scale is also dynamic, with a total of 273,469 pedestrian samples. Then we use Photoshop to synthesize rainy scenarios, with noise ratios of 5–35%, 35–65%, 65–95%, and fuzzy pixel values of 5–10, 10–15, and 15–20, respectively [8]. The synthetic image for the three rain conditions is shown in Figure 9.

### 4.2. Training Details

Experiments selected the mainstream deep learning framework PyTorch 3.5 as the experimental platform. The training environment is Intel Xeon Glod 5217 CPU 3 GHz, 64 GB memory, Nvidia TITAN RTX 24 G, Ubuntu 18.04, 64-bit operating system. The batch size of the proposed algorithm training process is 4, the learning rate is 0.0001, the input size of the network is 511 × 511, and the output size is 64 × 64.

### 4.3. Results on the Synthetic Datasets

We first use peak signal to noise ratio (PSNR) and structure similarity index (SSIM) to compare the quantitative de-raining performance of the de-raining module [6]. Quantitative results corresponding to different methods are tabulated in Table 1. It can be clearly seen that the de-raining module in this paper can achieve excellent de-raining performance. At a synthetic pedestrian dataset of rainy scenarios, the average PSNR and SSIM are up to 28.31 and 0.838 respectively. However, we find that the performance of de-raining is not the best for images in light rain. Because there are few noises in the image, the recurrent neural network cannot find the affected areas.

**Figure 10:**
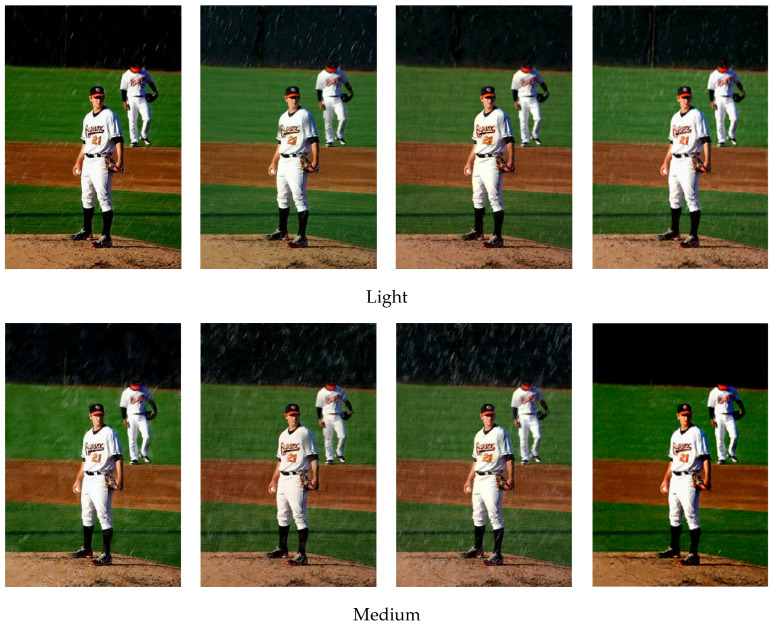

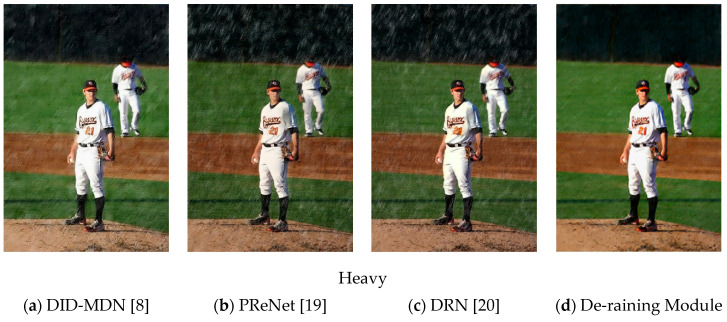
Rain-streak removal results on sample images from the synthetic datasets.

To visually demonstrate the improvements obtained by the de-raining module on the synthetic dataset, results on the sample image is presented in Figure 10. The de-raining module performs better on the image of medium and heavy rain. The pedestrians covered by rain streaks can be detected after processing. Previous methods tend to de-rain under specific rainy conditions. In contrast, the proposed method is able to deal with different types of rain conditions. In addition, it can be observed that the proposed method achieves better results in terms of effectively removing rain streaks while preserving the image details. However, it performs worse than DRN in light rain image processing and leaves rain streaks. In general, the de-raining performance of our de-raining module is better than the recent state-of-the-art de-raining methods.

Then, we compare pedestrian detection module with the state-of-the-art algorithm in terms of the performance of pedestrian detection. We use average precision (AP), average precision for the large objects (AP^l^), average precision for the medium objects (AP^m^), and average precision for the small objects (AP^s^) to evaluate the performance [41]. The results are shown in Table 2. The overall detection accuracy of this algorithm is better than YOLOv4. However, the accuracy of pedestrian detection is not the best for the medium target.

Since YOLOv4 is better than CornerNet-Lite in terms of the performance of pedestrian detection, we train YOLOv4 by the synthetic dataset on rainy days and compare it with our proposed algorithm that a pedestrian detection algorithm with a de-raining module. The specific test results are shown in Table 3.

From Table 2 and Table 3, we can find that the proposed algorithm restores the detection accuracy of the original image. For the scenarios of light, medium, and heavy rain, the proposed algorithm increases AP by 21.1%, 48.1%, and 60.9%. The results also show the necessity of the de-raining module. Previous methods could not accurately detect pedestrians even through the training of pedestrian datasets on rainy days. In contrast, our method can accurately detect pedestrians on rainy days.

**Figure 11:**
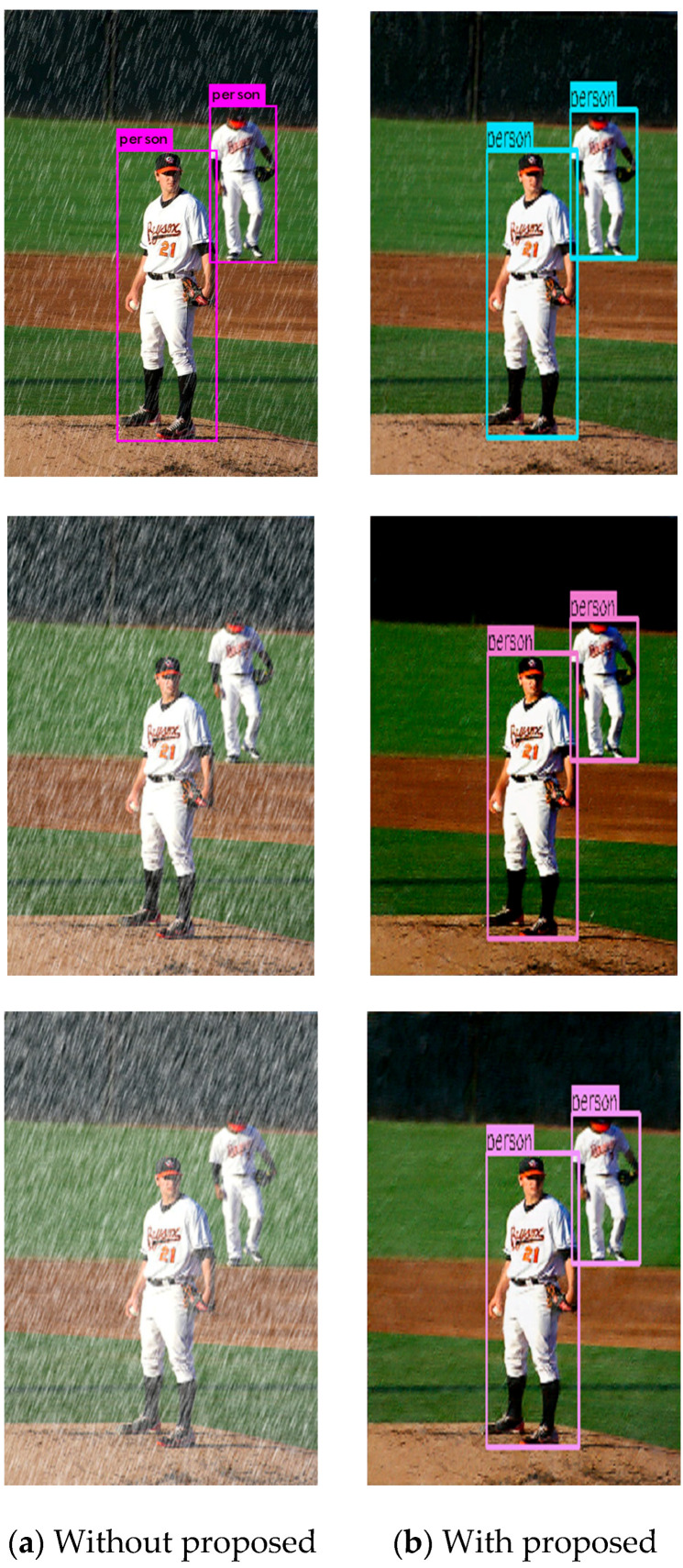
Qualitative sample comparison of the proposed algorithm.

To visually demonstrate the detection effect of this algorithm on the pedestrian dataset in rainy days, several representative pedestrian images are found from the test set. From top to bottom, the conditions of rain are light, medium, and heavy, as shown in Figure 11. We can easily observe that YOLOv4 cannot detect any pedestrian whether the rain is medium or heavy; in contrast, our proposed algorithm can detect all the pedestrians in the rainy images. Therefore, the proposed algorithm can realize pedestrian detection on rainy days with high detection accuracy.

### 4.4. Results on the Real-World Images

**Figure 12:**
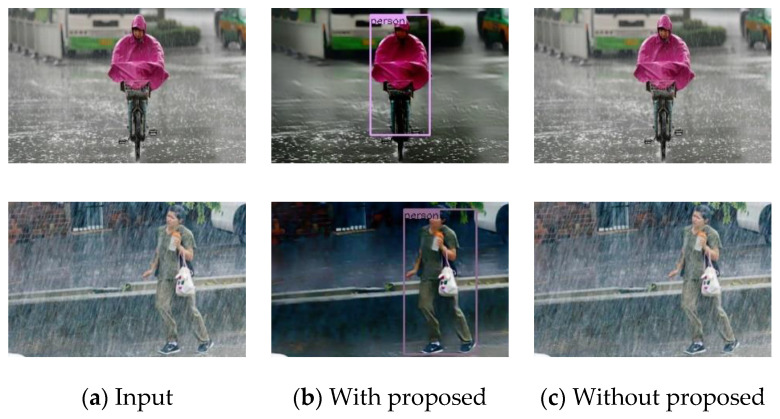
Pedestrian detection results on the real-world rainy images.

The performance of the proposed algorithm is also evaluated on many real-world images downloaded from the Internet. The test set has 30 images, and they are manually labeled by the software “labelme”. Then we generate a json file for testing. The de-rained results are shown in Figure 12. At the same time, we compare our proposed algorithm with YOLOv4 trained with synthetic rain dataset. It can be observed that the algorithm effectively removes rain streaks and accurately detects pedestrians while maintaining image details. In contrast, YOLOv4 did not detect pedestrians in moderate and heavy rain.

Also, the proposed algorithm can deal with different types of rain conditions, such as the medium rain shown on the first line of Figure 12 and the heavy rain shown in the second line of Figure 12. On the whole, the evaluation results of the real images show the effectiveness and robustness of the method.

## 5. Conclusions

The application of pedestrian detection in cooperative vehicle infrastructure systems is becoming more and more mature, but its detection performance on rainy days is not good. Rain streaks accumulate seriously and affect the visibility of the camera. In addition, most pedestrians on rainy days wear raincoats or carry umbrellas, so there is a lot of occlusions among pedestrians. In view of the particularity of pedestrian detection on rainy days, this paper proposed a novel algorithm with a de-raining module for detecting pedestrians. Compared with the existing pedestrian detection, this algorithm not only classifies the rainfall level of the image, but also effectively removes the rain streaks in the image and detects pedestrians. For the scenarios of light, medium, and heavy rain, extensive experiments on synthetic datasets demonstrate that the proposed algorithm increases AP of pedestrian detection by 21.1%, 48.1%, and 60.9%. The algorithm also performs well on real datasets. Moreover, the proposed algorithm achieves significant improvements over the recent state-of-the-art methods YOLOv4. In near future, we plan to optimize the parameters of this algorithm and study its performance in more complex environments.

## Figures and Tables

**Table 1:** Average PSNR and SSIM comparison on the synthetic datasets.

Conditions	Light		Medium		Heavy		Average	
Measure	PSNR	SSIM	PSNR	SSIM	PSNR	SSIM	PSNR	SSIM
DID-MDN [8]	29.86	0.899	25.9	0.713	25.3	0.755	27.02	0.789
PReNet [19]	29.42	0.892	24.9	0.698	15.07	0.542	23.13	0.711
DRN [20]	**30.78**	**0.937**	23.35	0.661	16.95	0.582	23.69	0.727
De-raining Module	30.56	0.921	**26.56**	**0.757**	**27.8**	**0.837**	**28.31**	**0.838**

**Table 2:** Comparison of pedestrian detection algorithms on original images

Algorithms	AP	AP^s^	AP^m^	AP^l^
CornerNet-Lite [30]	40.4	12.3	37.6	63.1
YOLOv4 [28]	42.1	12.7	**43.5**	62.6
Pedestrian Detection Module	**43**	**13.4**	39.3	**67.2**

**Table 3:** Average detection precision comparison between YOLOv4 and the proposed algorithm

Conditions	Without Proposed	With Proposed
Light	34.2	**41.4**
Medium	26.8	**39.7**
Heavy	23.5	**37.8**

## Data Availability

Publicly available datasets were analyzed in this study. This data can be found here: https://github.com/Liuyh2433209777/dataset.
